# Study on Mechanical Response and Structural Combination Design of Steel Bridge Deck Pavement Based on Multi-Scale Finite Element Simulation

**DOI:** 10.3390/ma19030448

**Published:** 2026-01-23

**Authors:** Jiping Wang, Jiaqi Tang, Tianshu Huang, Zhenqiang Han, Zhiyou Zeng, Haitao Ge

**Affiliations:** 1 School of Highway, Chang’an University, Xi’an 710064, China; huanglianhua139@126.com (J.W.); 2023221244@chd.edu.cn (J.T.); jtdongye@163.com (Z.Z.); haitao.ge@etu.unistra.fr (H.G.); 2 Guangxi Rongwu Expressway Co., Ltd., Wuzhou 543300, China; 19977194718@163.com; 3 Guangxi Dongye Expressway Co., Ltd., Nanning 530000, China; 4 School of Future Transportation, Chang’an University, Xi’an 710064, China

**Keywords:** multi-scale modeling, steel bridge deck pavement, structural configuration, finite element method, road engineering

## Abstract

Steel bridge deck pavements (SBDPs) are susceptible to complex mechanical and service environmental conditions, yet current design methods often struggle to simultaneously capture global bridge system behavior and local pavement responses. To address this issue, this study develops a multi-scale finite element modeling framework that integrates a full-bridge model, a refined girder-segment model, and a detailed pavement submodel. The framework is applied to an extra-long suspension bridge to evaluate the mechanical responses of five typical pavement structural configurations—including double-layer SMA, double-layer Epoxy Asphalt (EA), EA-SMA combinations, and a composite scheme with a thin epoxy resin aggregate overlay. By coupling global deformations from a full-bridge model to the local pavement submodel, the proposed method enables a consistent assessment of both bridge-level effects and pavement-level stress concentrations. The analysis reveals that pavement structures significantly alter the stress and strain distributions within the deck system. The results indicate that while the composite configuration with a thin overlay effectively reduces shear stress at the pavement–deck interface, it results in excessive tensile strain, posing a high risk of fatigue cracking. Conversely, the double-layer EA configuration exhibits the lowest fatigue-related strain, demonstrating superior deformation coordination, while the optimized EA-SMA combination offers a robust balance between fatigue control and interfacial stress distribution. These findings validate the effectiveness of the multi-scale approach for SBDP analysis and highlight that rational structural configuration selection—specifically balancing layer stiffness and thickness—is critical for enhancing the durability and long-term performance of steel bridge deck pavements.

## 1. Introduction

As an integral component of highways, bridges play a crucial role in ensuring smooth and reliable traffic. With the rapid development of China’s economy, numerous long-span steel bridges along rivers and coastal regions have been constructed or are under active construction, which has greatly promoted regional economic and social development. As an indispensable structural system, the bridge deck pavement is essential for protecting the deck plate, enhancing its stiffness, distributing vehicle loads, and extending the service life of the bridge deck [1,2,3]. However, the design service life of long-span steel bridges typically exceeds 100 years, while the deck pavement is much more susceptible to vehicle loading and environmental effects, resulting in a service life far shorter than that of the steel deck itself [4,5,6]. From the perspective of pavement design, on the one hand, bridge engineers and researchers tend to focus on the design of the bridge structure, emphasizing improvements to existing analysis methods and the development of new theoretical approaches to meet the rapidly evolving requirements of bridge structures, whereas relatively less attention is paid to deck pavement. On the other hand, bridge deck pavement design cannot be simply categorized as conventional highway pavement design, as there are significant differences between bridge deck pavement and ordinary road pavement structures in terms of material properties, dynamic characteristics, interlayer bonding, fatigue life, temperature response, and mechanical behavior [7]. More importantly, in contrast to the well-established elastic layered system theory for asphalt pavements and plate-on-elastic-foundation theory for cement concrete pavements, there is currently no standardized design procedure or unified evaluation criteria in the field of bridge deck pavement design and assessment.

At present, in order to obtain a clearer understanding of the mechanical response of bridge deck pavements, researchers commonly adopt the finite element method (FEM) to achieve relatively efficient and accurate design and evaluation of deck systems [8,9]. Based on mechanical analysis of the pavement system and appropriate mechanical performance indicators, mechanics-based approaches are used to guide structural design and material selection so that the composition and thickness of each layer can satisfy the requirements of coordinated deformation between the pavement and the steel bridge deck. Existing finite element analyses for orthotropic steel decks mainly include three categories: global model methods, submodel methods, and local simplified model methods. In the global model method, a full-bridge or full-span three-dimensional solid model including the pavement layers is directly established, and the overall response of the deck pavement under vehicle loads and temperature actions is obtained. For example, Zhao et al. [10] developed a three-span steel–concrete composite continuous girder global model to analyze the dynamic displacement and stress field of asphalt pavement; Song et al. [11] applied a full-bridge model to derive the nominal tensile stress in an LUHPC overlay for fatigue design. Such approaches can fully account for the spatial coupling among main girders, towers, hangers, and other structural members, as well as the global dynamic interaction among vehicles, bridges, and pavement. They are therefore suitable for evaluating the overall influence of different bridge types, span arrangements, and load combinations on the stress state of the pavement layer. However, due to the very large number of degrees of freedom, the pavement layer in the global model can hardly be meshed with very fine elements or assigned highly sophisticated constitutive models; as a result, interface behavior and local stress concentrations are difficult to capture in detail, and the computational cost of parametric sensitivity analyses and multi-scenario simulations becomes high.

The local simplified model method, to some extent, discards the overall bridge structure and considers only a representative region of the orthotropic steel deck and its pavement for analysis, typically assuming simplified boundary constraints and loading conditions. For instance, in developing an XGBoost-based performance prediction model for steel bridge deck pavements, Wei et al. [12] employed a local finite element model of 4.2 m × 9.0 m as the computational basis, which included several U-shaped stiffeners and diaphragms; shell elements were used for the steel deck and stiffeners and solid elements for the pavement layers. By applying wheel loads at critical positions, key stress and strain indices were obtained and then used to generate datasets for training a machine learning model. In a review of finite element methods for steel bridge deck pavements, Sun et al. [13] classified such models that contain only the steel deck and pavement as “local/simplified model methods” and pointed out that they offer significant advantages in modeling simplicity and computational efficiency for parametric analyses and structural scheme comparison. Some studies [14,15] have gone a step further by simplifying local three-dimensional models into two-dimensional composite beam models or mesoscale simplified models and solving the responses under dynamic loads using analytical or semi-analytical approaches, thereby achieving higher efficiency in large-scale parametric sweeps and sensitivity analyses. Nevertheless, because simplified models cannot accurately reflect global effects such as main girder constraints, span arrangements, and temperature gradients, their results are highly dependent on boundary assumptions and may underestimate or overestimate stress levels under specific load cases. Min et al. [16], in their study on rutting mechanisms of steel bridge decks, also explicitly noted that mesoscale simplified models cannot fully capture full-bridge behavior and should therefore be used with caution in interpreting results.

The submodel method is constructed on the basis of a global bridge or girder-segment coarse model, within which the steel box girder, orthotropic steel deck, and pavement layers in critical regions are modeled in a refined manner. The boundary responses obtained from the global or girder-segment model are then transferred to the submodel as boundary conditions, thereby combining the bridge system effects with high-resolution local stress analysis of the pavement. Nie et al. [17], for example, extracted displacements and internal forces at key sections from a full-bridge finite element model and imposed them on a local submodel including the orthotropic steel deck and epoxy asphalt pavement to compute pavement strain levels and compare them with material fatigue limits, thus achieving an integrated structure–material design. Chen et al. [18,19] proposed a multi-scale modeling framework in which global, segmental, and local submodels are linked in series to investigate fatigue cracking, primarily focusing on the negative bending moment regions (e.g., 1/4 span) where surface tensile stress is predominant. Zhang et al. [20], focusing on a multi-tower suspension bridge, introduced stochastic traffic loads and used a hierarchical global-to-local modeling system based on internal force transmission (bending and torque) near tower sections to analyze the dynamic response and fatigue damage evolution. However, these studies often prioritize stress-controlled negative moment zones, potentially overlooking the complex pavement mechanics induced by large-deformation positive bending moment regions in long-span suspension systems. The submodel method has demonstrated substantial advantages in finite element analysis of steel bridge deck pavements: on the one hand, it introduces bridge system effects through the upper-level model, avoiding the neglect of global structural behavior inherent in purely local analyses; on the other hand, the submodel can employ combined shell–solid elements, viscoelastic–viscoplastic constitutive models, and contact/cohesive-zone models at interfaces to finely simulate the temperature sensitivity of asphalt layers, interlayer sliding, and crack propagation.

In terms of structural configurations, current steel bridge deck pavements are mainly developed around several typical combinations, such as double-layer epoxy asphalt concrete (EA–EA), epoxy asphalt as the lower layer with SMA as the upper layer (EA–SMA), and double-layer SMA (SMA–SMA). These structural forms have evolved into relatively mature families through patents and engineering practice. To select the most suitable deck pavement for a given bridge, a large number of mechanical analyses and material tests are usually required [21,22,23]. However, existing studies on structural scheme comparison still suffer from two major limitations. First, most finite element models are constructed at a single scale, either as global models or local simplified models, and often use the maximum tensile stress or rutting deformation at a single section as the evaluation criterion. This makes it difficult to simultaneously capture bridge system effects and local stress concentrations in a unified framework, and the responses at sensitive areas such as interlayer interfaces and rib-to-deck regions cannot be described in sufficient detail [24]. Second, the comparison of structural schemes is often highly tied to specific projects or experimental setups, with evaluation indicators focusing on a single load case or a single performance aspect (e.g., high-temperature stability or a particular fatigue life). Systematic comparisons under multiple temperatures, multiple load spectra, and multiple response indicators (tensile stress, shear stress, rutting, interlayer sliding, etc.) are lacking, which makes it difficult to quantitatively assess the relative merits of different structural types under comparable conditions [25].

In this context, the present study conducts a comparative analysis of steel bridge deck pavement structural schemes based on a multi-scale modeling framework. Specifically, the analysis targets the mechanical response characteristics of the pavement within the critical positive bending moment girder segment. While it is acknowledged that cross-sections with the maximum curvature (typically located in negative bending regions) often exhibit high strains, the local bending effect induced by wheel loads in these regions may partially counteract the global deformation background. In contrast, within the positive bending region characterized by maximum vertical deflection, the wheel load effect is more likely to superpose with the global downward deflection, creating a potentially more critical combined loading condition. Furthermore, compared to the extensively investigated negative bending regions, the pavement response at the maximum-deflection section in positive bending regions remains less explored. Therefore, the cross-section exhibiting the maximum vertical deflection under the most unfavorable global loading condition is selected as the representative target. Under boundary conditions governed uniformly by this global deflection, multi-indicator mechanical responses at sensitive locations are systematically computed to facilitate quantitative comparison and optimization. Through this multi-scale analysis, which simultaneously accounts for the deformation effects of the bridge system and local stress accuracy, the study aims to provide a quantifiable mechanical basis for the selection and design of long-life steel bridge deck pavement structures.

## 2. Finite Element Model Development and Structural Schemes

### 2.1. Global Bridge-Scale Model and Deformation Analysis

#### 2.1.1. Project Background and Parameters

The Xunjiang Extra-Long Bridge is located on the Wuzhou–Yulin–Qinzhou Expressway (Cangwu–Rong County section), crossing the Xunjiang River within Teng County, Wuzhou City, with a total length of 1688 m. The main bridge adopts a steel box girder suspension bridge system, while the approach bridges are designed as reinforced concrete T-girder bridges.

**Figure 1:**
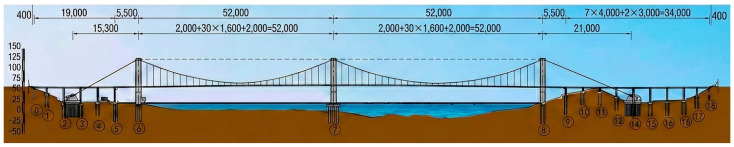
Bridge Model Parameters (Unit: cm).

The main bridge is configured as a two-span suspension bridge with a steel box girder deck and a span arrangement of 2 × 520 m. The main cables are arranged in a spatial cable system, and the overall span layout is 153 + 2 × 520 + 200 m, with a rise-to-span ratio of 1/9.16. Each main span is equipped with 31 pairs of hangers, with a longitudinal spacing of 16 m between adjacent hangers. The structural parameters are shown in Figure 1.

#### 2.1.2. Development and Analysis of the Full-Bridge FEM in MIDAS Civil

Considering the pronounced geometric nonlinearity of kilometer-scale suspension bridges, the global deformation is transmitted through the steel box girder to the bridge deck pavement, resulting in a highly non-uniform stress state. In this study, a refined full-bridge finite element model incorporating the main cables, hangers, and steel box girder was established on the MIDAS Civil platform. By simulating a series of static and dynamic loading conditions, the regions with extreme displacement and deformation along the entire bridge were identified, providing accurate spatial locations and deformation magnitudes for subsequent locally refined modeling.

**Figure 2:**
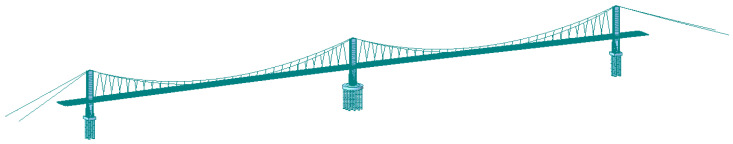
Schematic diagram of the MIDAS finite element model.

During the modeling process, only the main girder of the suspension bridge segment was considered. A complete numerical model was constructed in MIDAS according to the actual arrangement of the main cables and hangers. The boundary conditions were defined with full consideration of the bearing configuration at the junction between the main bridge and the approach spans, as well as the contact boundary conditions between the main girder and the central and side towers. At the central tower, transverse wind-resistant bearings and longitudinal restraining bearings were applied, whereas at the side towers, transverse wind-resistant bearings were installed. In the transition regions, vertical bearings together with transverse wind-resistant bearings were provided. A schematic of the established suspension bridge model is shown in Figure 2.

**Figure 3:**
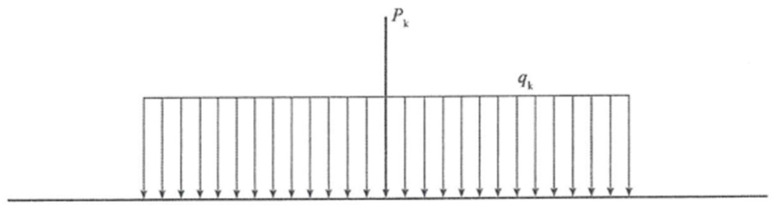
Schematic diagram of lane loading.

According to the *General Code for Design of Highway Bridges and Culverts* [26], the load types considered in the analysis of the main bridge primarily include permanent actions, variable actions, and accidental actions. In this study, the self-weight of the main girder and the weight of the deck pavement are taken as permanent loads, where the unit weight of steel is taken as 78.5 kN/m^3^ and that of the pavement is taken as 24 kN/m^3^. For variable actions, the standard Highway Class I vehicle load specified in the code is adopted as the lane load, with the uniformly distributed load q_k_ set to 10.5 kN/m and the concentrated load P_k_ taken as 360 kN according to the bridge span (see Figure 3). In addition, wind loads and temperature effects of the main girder are also considered as part of the variable actions, while accidental actions are not included at this stage. Based on the combined effect of permanent and variable loads, a stress and displacement–deformation analysis model of the suspension bridge under different loading conditions is established.

**Figure 4:**
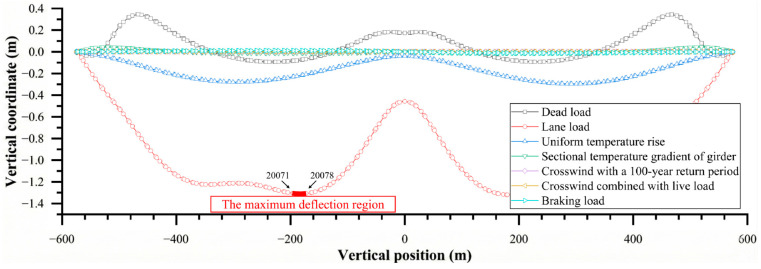
Global displacement and deformation of the bridge.

Using MIDAS Civil, several code-specified load combinations—including dead load, lane load, temperature rise, and cross-wind—were applied to the model to simulate different service conditions of the bridge during actual operation. For each load combination, the global displacement and deformation distribution of the entire bridge was computed (see Figure 4).

The analysis results indicate that, under the action of lane loading, the longitudinal displacement and deformation of the main girder reach their maximum values, identifying this condition as the most unfavorable load case. Further investigation shows that the maximum longitudinal displacement of the main girder occurs at a location approximately 180 m from the mid-span toward the Cangwu side, where the downward deflection reaches 1.31 m. This section is therefore selected as the critical cross-section for assessing girder deformation.

### 2.2. Development of a Cross-Scale Coupled Model Between the Global Bridge and the Local Girder Segment

Based on the global deformation data obtained from MIDAS Civil in the previous section, it is noted that MIDAS Civil has limitations in modeling complex material behaviors such as asphalt mixtures, whereas ABAQUS 2023 offers significant advantages in simulating nonlinear responses, multi-physics coupling, and highly customized analysis. Therefore, ABAQUS is adopted for the subsequent detailed analysis. Using the full-bridge deformation results as input, a local submodel of the main girder under the most unfavorable global deformation condition is established in ABAQUS, which serves as the fundamental model for the simulation analysis of the bridge deck pavement.

#### 2.2.1. Construction of the Local Girder Submodel and Implementation of Cross-Scale Boundary Conditions

**Figure 5:**
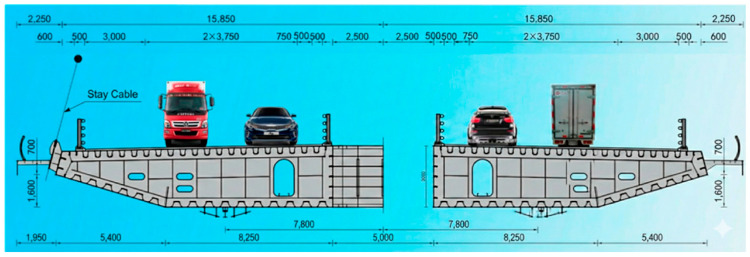
Standard cross-section of the main girder (Unit: mm).

According to the relevant design documents, the section where the main girder exhibits the most severe deformation is located within the B-type girder segment (i.e., the standard segment), whose design parameters are shown in Figure 5. To accurately characterize the local deformation behavior of the girder under the most unfavorable load condition, a refined local submodel is constructed in ABAQUS based on the design parameters of the B-type segment. This submodel is used to simulate the stress, displacement, and structural response of the B-type segment under adverse deformation, providing essential data support for subsequent bridge design optimization and durability assessment.

Considering the structural characteristics of the steel box girder, the main girder consists of two approximately symmetric steel box girders connected by transverse diaphragms. To improve computational efficiency, the steel box girder is simplified in this study by modeling only half of the cross-section, and symmetry boundary conditions are applied at the transverse connection boxes to represent its actual mechanical behavior.

According to the global displacement analysis in Figure 4, the steel box girder exhibits a pronounced initial deformation under the lane load. To simultaneously capture the bridge system effect and the refined response of the local deck pavement, a multi-scale submodeling framework of “full bridge–girder segment–deck pavement” is adopted in this study. The basic idea is as follows: the global bridge model is first used to obtain the overall longitudinal deformation of the main girder under realistic load combinations, and the corresponding displacement field is then transferred to the boundaries of the local steel box girder–pavement model, so that the global internal force and deformation state are implicitly introduced into the local model. This strategy is consistent with the multi-scale approaches proposed by Nie et al. and Chen et al. [17,18,19], in which the displacement response of the upper-level model is employed as the boundary condition of the lower-level model, and it allows the mechanical state of the steel bridge deck pavement within the overall stress field to be represented with higher fidelity without a significant increase in computational cost.

The length of the local steel box girder–pavement submodel is set to 16 m. On the one hand, this interval covers the maximum deflection region and its adjacent transition zones; on the other hand, it includes two cross-beams and several U-shaped ribs, ensuring that the central area is not affected by end truncation effects. The deformation curve of the corresponding box-girder segment in the full-bridge model is extracted, and the nodal displacement data of several elements in the vicinity of the maximum deflection region are summarized in Table 1.

Using these data pairs—girder node coordinates X (in m) and corresponding vertical deformation Y (in m)—a set of characteristic points (X_i_, Y_i_) is obtained from the global finite element results. A least-squares polynomial fitting is then performed:

(1) $$w\left(x\right)=a_{0}+a_{1}x+a_{2}x^{2}+\ldots +a_{n}x^{n},$$

**Figure 6:**
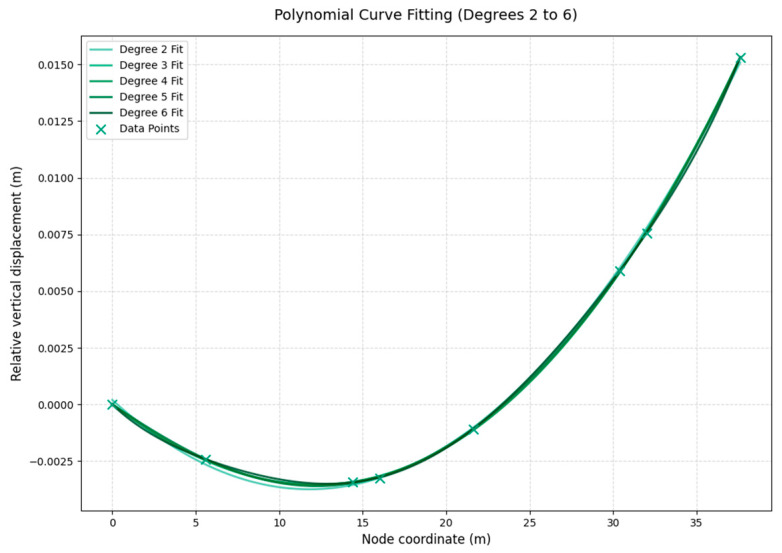
Fitted deformation curve of the main girder near the maximum deflection under the most unfavorable load case.

Polynomial orders from n = 3 to n = 6 are tested, and the residuals between the fitted curves and the discrete finite element data are compared. The results indicate that the sixth-order polynomial yields smaller mean-square errors than lower-order polynomials and can satisfactorily capture the nonlinear deformation characteristics in the vicinity of the maximum deflection region of the main girder. Therefore, the sixth-order polynomial is finally adopted as the continuous representation of the initial deformation of the main girder (see Figure 6). Based on the fitted curve in the maximum deflection region, the peak vertical deformation occurs at the coordinate x = 12.88 m, with a magnitude of approximately 3.5 mm relative to the origin.

**Figure 7:**
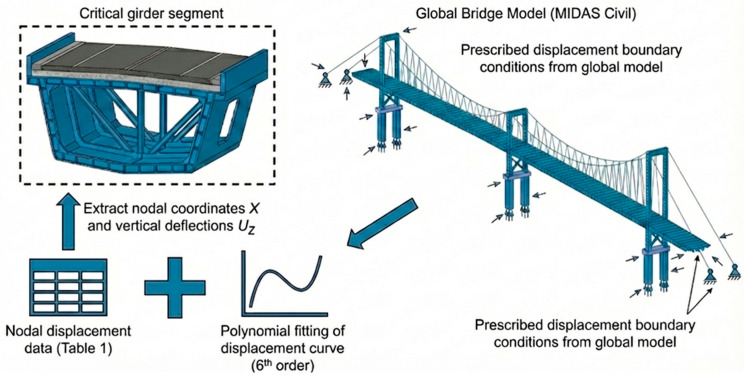
Transfer of Global Boundary Conditions to Local Submodel.

**Figure 8:**
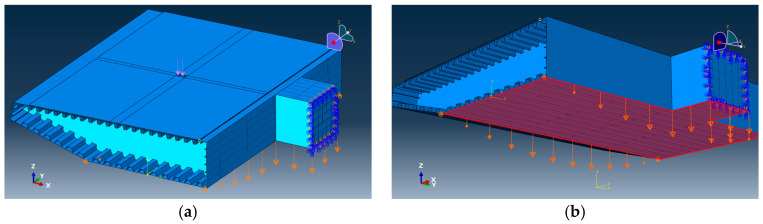
Boundary condition settings of the coupled steel box girder–pavement model. (**a**) Local steel box girder–pavement coupled model geometry and constrained boundaries; (**b**) Imposed displacement boundary conditions (highlighted) for introducing the global deflection field.

For the submodel, the suspension-bridge steel box girder in this segment is subject to complex boundary conditions, including displacement deformation, torsion, in-plane distortion, and warping. However, under the large deformation of the steel box girder induced by the lane-load case, the stress/strain state of the pavement layer is primarily driven by the deformation of the steel deck plate in direct contact with the pavement. Within this research framework, transferring the vertical deflection field obtained from the global bridge analysis to the submodel boundaries and prescribing the corresponding vertical displacement constraints on the steel box girder is sufficient for a comparative assessment of the deck pavement responses (see Figure 7). Therefore, according to this deformation curve, the corresponding displacement boundary conditions are imposed on the bottom surface of the steel box girder–pavement coupled model in ABAQUS (see Figure 8) to introduce the initial deflection. A comparison between the deflection curve of the local model under only the initial deformation and that of the corresponding segment in the full-bridge model shows that the difference in maximum deflection is within 5%, indicating that the equivalent boundary settings can satisfactorily reconstruct the mechanical and deformation state of the critical girder segment within the full-bridge system.

In the refined local steel box girder–deck pavement coupled model, both the steel box girder and the pavement layers were discretized using three-dimensional solid elements. The steel box girder was modeled with C3D10 tetrahedral elements with a relatively coarse mesh (as the detailed mechanical response of the steel box girder itself is not the primary focus of this study), whereas the deck pavement was modeled with C3D8R reduced-integration hexahedral elements with a relatively dense mesh to ensure adequate resolution of the key pavement responses.

Regarding boundary conditions, displacement constraints were applied at the four bottom corner points to restrain the X and Y-direction translations (the Z-direction displacement had already been prescribed by the transferred displacement boundary condition), thereby eliminating rigid-body motions and allowing the edge rotations to develop naturally under the imposed Z-direction displacement field. Considering the transverse symmetry at the box-section connection plane, a symmetry boundary condition with the X-axis as the plane normal was imposed at this section, i.e., constraining the X-direction displacement and the rotations about the Y and Z-axes, so that the mechanical response of the double-sided steel box girder could be represented even though only one side was explicitly modeled.

For the interlayer/interface definition, epoxy resin is used in practice as the bonding agent between asphalt layers and between the pavement system and the steel deck plate, providing a high bonding strength. Therefore, no relative slip was assumed at the asphalt interlayers or at the pavement–deck interface, and tie constraints were adopted.

In terms of solver settings, a static analysis step was used with geometric nonlinearity deactivated. The total step time was set to 1, and the initial increment was set to 0.2, which allows the analysis to be completed in approximately four increments under the default ABAQUS increment strategy.

After establishing the local steel box girder–pavement coupled model, the initial deflection of the main girder obtained from the global analysis is first applied via displacement boundary conditions, so that the model is in a deformation equilibrium state under dead load and lane load before the wheel load is introduced, the entire process is illustrated in the figure. Subsequently, a standard dual-circular, uniformly distributed vertical load with uniform pressure is superimposed in the central region of the deck pavement, which is equivalent to considering the additional effect of a single vehicle on the most unfavorable internal-force state and thus yields more conservative key responses of the deck pavement. The load application position is taken at the center of the deck pavement, which is farthest from the truncated boundaries, thereby minimizing the influence of boundary constraints; under wheel loading, this location typically develops the largest longitudinal tensile strain along the traffic direction. The load form follows the dual circular uniformly distributed load specified in the design code, as shown in Figure 8a. The design axle load parameters [27] are listed in Table 2.

#### 2.2.2. Configuration of Pavement Structural Schemes and Material Parameters

After the steel box girder is fully modeled, the bridge deck pavement layers are then constructed on its top surface, and the cross-scale boundary conditions are applied subsequently. In current engineering practice, commonly used deck pavement materials include dense-graded asphalt concrete (AC), stone mastic asphalt (SMA, PSMA), gussasphalt concrete (GA, GAM), and epoxy asphalt mixtures (EA), among others. Among these, SMA-type pavements are widely used in various structural schemes, while the appropriate use of EA can significantly reduce the cracking risk of the deck pavement.

**Figure 9:**
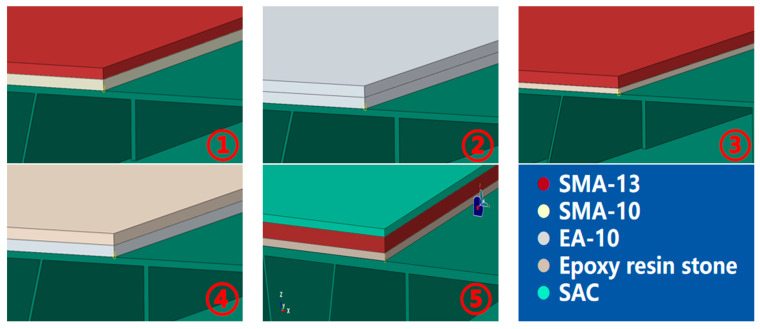
Five types of ABAQUS steel box girder–pavement coupled models.

To investigate the performance of different paving materials, including SMA and EA, the equivalent elastic modulus for the asphalt layers was determined based on the compressive resilient modulus measured in laboratory tests at 30 °C. Based on the design scheme and actual engineering conditions, five typical bridge deck pavement structural configurations are proposed (see Table 3). Correspondingly, five coupled finite element models of the steel box girder–deck pavement system are established according to the material types and structural schemes (shown in Figure 9), with the material parameters obtained from the tests listed in Table 4.

## 3. Analysis of the Mechanical Response of Steel Bridge Deck Pavement Structures Under Loading

**Figure 10:**
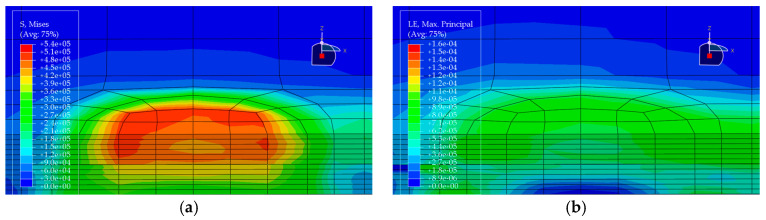
Stress and strain contour plots of Structural Scheme 1. (**a**) Stress contour plot; (**b**) Strain contour plot.

**Figure 11:**
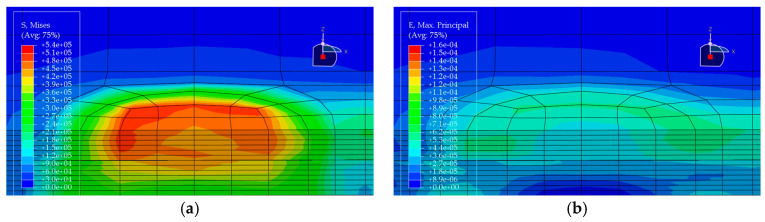
Stress and strain contour plots of Structural Scheme 2. (**a**) Stress contour plot; (**b**) Strain contour plot.

**Figure 12:**
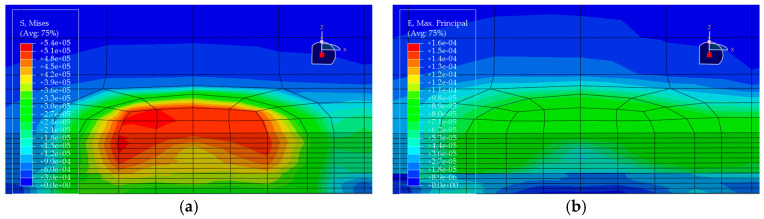
Stress and strain contour plots of Structural Scheme 3. (**a**) Stress contour plot; (**b**) Strain contour plot.

**Figure 13:**
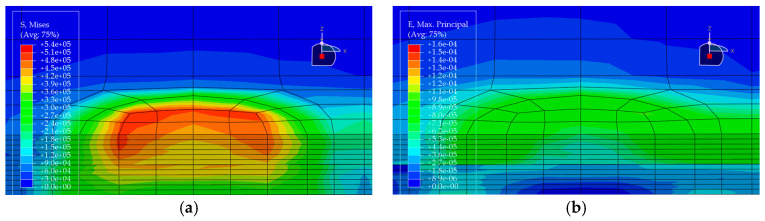
Stress and strain contour plots of Structural Scheme 4. (**a**) Stress contour plot; (**b**) Strain contour plot.

**Figure 14:**
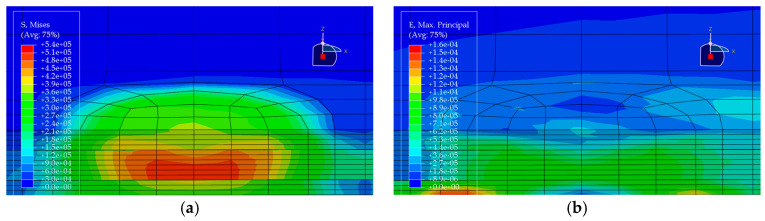
Stress and strain contour plots of Structural Scheme 5. (**a**) Stress contour plot; (**b**) Strain contour plot.

Under the standard axle load condition, the same load is applied to each of the five pavement structures, and the corresponding stress and strain distributions are obtained (see Figure 10, Figure 11, Figure 12, Figure 13 and Figure 14). In each figure, (a) shows the stress contour plot and (b) shows the strain contour plot, both extracted at the loading section to visually illustrate the distribution patterns. The maximum vertical tensile stress, longitudinal tensile strain at bottom of pavement layer, and interlayer shear stress responses of the bridge deck pavement under the standard axle load are summarized in Table 5.

Based on the finite element results of the five pavement structural schemes listed in Table 5, it is evident that the stress characteristics of each scheme within the steel bridge deck pavement system differ significantly.

From the viewpoint of global flexural response of the pavement, the maximum vertical tensile stress is highest in Scheme ② (35 mm EA-10 + 30 mm EA-10), reaching 0.06470 MPa, whereas Scheme ⑤ (15 mm Thin epoxy resin aggregate overlay + 30 mm SMA-10 + 20 mm SAC wearing course) exhibits a much lower value of only 0.02112 MPa. This corresponds to a reduction of approximately 67% relative to Scheme ② and about 56% relative to the all-SMA configuration of Scheme ①. This indicates that the introduction of an thin epoxy resin aggregate overlay together with a SAC wearing course enhances the overall flexural stiffness of the pavement system and leads to a noticeable redistribution of internal stresses, thereby effectively reducing peak tensile stresses within the pavement. The maximum vertical tensile stresses in Scheme ① (SMA-10 + SMA-13) and Scheme ④ (30 mm EA-10 + 30 mm SMA-10) fall in an intermediate range (0.04792–0.05001 MPa), both lower than that of the pure double-layer epoxy system in Scheme ② and the scheme with a pronounced stiffness gradient in Scheme ③.

Based on the finite-element results for the five deck pavement configurations in Table 5, a quantitative comparison and integrated assessment of their mechanical response characteristics were carried out. Regarding the key indicator associated with fatigue-related cracking behavior—namely the longitudinal tensile strain at the bottom of the pavement layer along the traffic direction—the five schemes exhibit pronounced differences. Scheme ② (35 mm EA + 30 mm EA) benefits from the relatively high modulus of the full epoxy asphalt concrete system, resulting in the lowest bottom tensile strain of only 1.24 με among all schemes, indicating the most effective deformation control in response to steel deck deformation and the most favorable fatigue-related strain demand. Scheme ④ (30 mm EA + 30 mm SMA) ranks second, with a tensile strain of 1.59 με, demonstrating the effectiveness of increasing the stiffness of the lower layer in the “stiff-bottom/soft-top” combination for limiting tensile deformation. In contrast, Scheme ⑤, which adopts an ultra-thin 15 mm epoxy resin aggregate overlay at the bottom, shows a markedly increased bottom tensile strain of 14.67 με, representing an abrupt escalation. This implies that such a thin layer is prone to excessive tensile deformation under heavy loading and may be associated with a substantially elevated fatigue-cracking susceptibility; without a specially engineered material with high ductility, this configuration is not ideal when the thin layer is intended to function as a primary load-carrying component.

Further examination of interlayer shear stresses and vertical tensile stress distributions provides insight into interlayer stability and potential debonding risk. For interlayer shear stresses between asphalt layers, Schemes ① and ② perform best, with the maximum shear stress controlled within 0.087 MPa, suggesting that equal-thickness and/or homogeneous-material systems are more conducive to a uniform transfer of interlayer shear. Notably, Scheme ③ (20 mm EA + 40 mm SMA) exhibits the peak transverse maximum shear stresses both at the asphalt interlayer and at the pavement–steel deck interface, reaching 0.1116 MPa and 0.1495 MPa, respectively, the highest among the five schemes. This disadvantage indicates that in a “stiff-bottom/soft-top” system, an excessively thin lower EA layer (20 mm) not only fails to provide an effective stiffness-transition and stress-dispersion function but may also lead to pronounced stress concentration, thereby increasing the likelihood of interlayer slippage and debonding. By comparison, Scheme ④, with the EA lower-layer thickness increased from 20 mm to 30 mm, shows effective reductions in both interface shear stress and bottom tensile stress, suggesting that the stiff lower layer has a thickness threshold that yields improved mechanical effectiveness.

Considering the maximum vertical tensile stress, although Scheme ② shows the most favorable fatigue-related strain demand, its maximum vertical tensile stress reaches 0.0647 MPa, the highest among all schemes. This imposes a higher requirement on the internal cohesion of the epoxy asphalt mixture to prevent internal tearing induced by vertical loading. Scheme ⑤, despite its unfavorable strain indicator, shows clear advantages in reducing the pavement–deck interface shear stress (0.1201 MPa) and the maximum vertical tensile stress (0.0211 MPa), indicating that thin-layer surfacing can contribute to lowering interface debonding-related stress demands under certain conditions. Overall, Scheme ② is suitable for bridges with heavy traffic loading where fatigue-related performance demands are stringent, but it requires strict construction quality control to resist vertical tensile action. Scheme ④ achieves a comparatively low tensile strain demand while avoiding shear-stress concentration, balancing mechanical performance and economy, and thus represents a more robust and well-balanced option. In contrast, Scheme ③ exhibits an evident drawback of shear-stress concentration and should be adopted with caution in engineering practice.

A comparative pros–cons assessment of the five steel bridge deck pavement schemes based on the FE response indicators is presented in Table 6.

## 4. Conclusions

This study establishes a multi-scale finite element modeling framework that couples global bridge response with detailed pavement-level analysis. By effectively coupling a global full-bridge model with a locally refined model, the overall deformation effects of the bridge system are introduced into the stress analysis of the pavement details, making the calculated stress–strain responses of the pavement layers more consistent with real service conditions. At the same time, the local model provides a detailed depiction of the internal stress state of different pavement structures, allowing for the identification of stress concentration regions and potential weak zones in each scheme. This macro–micro integrated analysis approach overcomes the limitations of single-scale models, which struggle to simultaneously capture both global behavior and local details, and significantly improves the accuracy and reliability of the analysis results.

Based on the modeling framework, this study conducts a comparative mechanical performance analysis of five typical steel bridge deck pavement structural schemes. The results indicate that different pavement configurations lead to pronounced differences in stress levels and deformation patterns, confirming that the pavement structure type has a significant influence on the mechanical behavior and durability of the system. Among the schemes, the double-layer epoxy asphalt configuration (Scheme ②) demonstrates superior capacity for coordinating deformation with the steel deck, exhibiting the lowest bottom tensile strain (1.24 με) and thus the highest potential resistance to fatigue cracking, although its higher vertical tensile stress necessitates robust material cohesion. In contrast, the composite scheme comprising an ultra-thin epoxy resin aggregate overlay (Scheme ⑤), while effective in minimizing pavement–deck interface shear and vertical tensile stresses (beneficial for mitigating debonding risks), exhibits an abrupt escalation in bottom tensile strain (14.67 με). This suggests that despite its interface advantages, Scheme ⑤ carries a significantly heightened susceptibility to fatigue cracking compared to the stiffer EA-based schemes. Furthermore, the analysis highlights the importance of layer thickness optimization; Scheme ④ (30 mm EA + 30 mm SMA) achieves a balanced performance by avoiding the shear stress concentrations observed in Scheme ③ (20 mm EA + 40 mm SMA) while maintaining a low strain level. These findings suggest that rational optimization—specifically balancing the stiffness transition and layer thickness—is critical for minimizing stress–strain responses and enhancing the long-term durability of steel bridge deck pavements.

Overall, the multi-scale modeling framework provides a systematic and reliable tool for comparing pavement structural schemes and supports informed decision-making in the design and optimization of steel bridge deck pavements. Future work will incorporate temperature, moisture, and the evolution of material properties under environmental loads to further improve its capability to simulate actual service environments and to provide long-term performance assessments of deck pavement.

## Figures and Tables

**Table 1:** Nodal deformation data of the full bridge under the most unfavorable load case.

Node ID	Node Coordinate (m)	Absolute Vertical Displacement (m)	Relative Vertical Displacement (m)
20071	0	−1.306176	0
20072	5.6	−1.308591	−0.002415
20073	14.4	−1.309595	−0.003419
20074	16	−1.30941	−0.003234
20075	21.6	−1.307254	−0.001078
20076	30.4	−1.300266	0.00591
20077	32	−1.298603	0.007573
20078	37.6	−1.290853	0.015323

**Table 2:** Design axle-load parameters [27].

Design Axle Load (kN)	Tire Contact Pressure (MPa)	Equivalent Circular Contact Diameter of Single Tire (mm)	Center-to-Center Spacing of Dual Tires (mm)
100	0.7	213.0	319.5

**Table 3:** Pavement structural configuration.

No.	Lower Layer	Upper Layer	Wearing Course
①	30 mm SMA-10	30 mm SMA-13	/
②	35 mm EA-10	30 mm EA-10	/
③	20 mm EA-10	40 mm SMA-13	/
④	30 mm EA-10	30 mm SMA-10	/
⑤	15 mm Thin epoxy resin aggregate overlay	30 mm SMA-10	20 mm SAC

**Table 4:** Material property parameters.

Parameter	EA-10	SMA-10	SMA-13	Epoxy Resin Stone	SAC	Steel Box Girder
Density (kg/m^3^)	2465	2460	2461	2120	2430	7850
Elastic modulus (MPa)	2400	1400	1600	600	1200	200,000
Poisson’s ratio	0.35	0.35	0.35	0.35	0.35	0.3

**Table 5:** Main stress analysis results of the five bridge deck pavement structures.

Scheme	Maximum Vertical Tensile Stress (MPa)	Longitudinal Tensile Strain at Bottom of Pavement Layer (×10^−6^)	Maximum Transverse Interlayer Shear Stress in Asphalt Layers (MPa)	Maximum Longitudinal Interlayer Shear Stress in Asphalt Layers (MPa)	Maximum Transverse Shear Stress at Pavement-Deck Interface (MPa)	Maximum Longitudinal Shear Stress at Pavement-Deck Interface (MPa)
①	0.04792	2.81262	0.08601	0.07585	0.1427	0.1353
②	0.06470	1.24051	0.08676	0.08104	0.1460	0.1372
③	0.05228	3.84498	0.1116	0.1018	0.1495	0.1413
④	0.05001	1.59348	0.1008	0.09230	0.1483	0.1413
⑤	0.02112	14.6673	0.1060	0.09647	0.1201	0.1147

**Table 6:** Comparative pros–cons assessment of five steel bridge deck pavement (SBDP) schemes based on FE response indicators.

Scheme	Structural Feature	Pros	Cons	Overall Assessment
①	Double-layer SMA	Low interlayer shear stress; relatively balanced load transfer.	Moderate bottom tensile strain; inferior fatigue-related strain control compared with EA-based schemes.	Suitable for conventional traffic levels with relatively high construction tolerance, but the fatigue-related performance may be less favorable than EA-based schemes.
②	Double-layer EA	Best fatigue-related strain control; good interlayer shear performance.	Highest maximum vertical tensile stress, requiring very high internal cohesion of the material; typically the highest cost.	Suitable for heavy-traffic bridges and cases where orthotropic-deck fatigue is a key concern; strict construction quality control is required to withstand vertical tensile action.
③	Thin EA + thick SMA	No pronounced mechanical advantage.	Highest risk of shear-stress concentration (peak shear at both interlayer and pavement–deck interface); relatively large bottom tensile strain.	A 20 mm EA layer may be too thin to provide an effective stiffness transition, leading to stress concentration; not recommended as a first-choice option.
④	Equal-thickness EA + SMA	Relatively low bottom tensile strain (second only to double EA), balancing fatigue-related strain control and cost.	Interface shear stress is slightly higher and close to Scheme ③.	Achieves a good balance between fatigue-related strain demand and cost; a 30 mm EA layer provides a more effective stiffening/transition effect than 20 mm.
⑤	SAC + SMA + Thin epoxy resin aggregate overlay	Lowest pavement–deck interface shear stress (lower debonding-related demand); lowest maximum vertical tensile stress.	Extremely high bottom tensile strain, implying a very high fatigue-cracking susceptibility.	Although interface responses are favorable, the extremely high tensile strain requires the epoxy resin aggregate overlay to have very high tensile strain capacity; otherwise, reflective cracking is likely to occur.

## Data Availability

The original contributions presented in this study are included in the article. Further inquiries can be directed to the corresponding author.
